# *Drosophila* immunity: the *Drosocin* gene encodes two host defence peptides with pathogen-specific roles

**DOI:** 10.1098/rspb.2022.0773

**Published:** 2022-06-29

**Authors:** M. A. Hanson, S. Kondo, B. Lemaitre

**Affiliations:** ^1^ Global Health Institute, School of Life Science, École Polytechnique Fédérale de Lausanne (EPFL), Lausanne, Switzerland; ^2^ Invertebrate Genetics Laboratory, Genetic Strains Research Center, National Institute of Genetics, Mishima, Japan

**Keywords:** antimicrobial peptide, host–pathogen interactions, *Drosophila*, immunity

## Abstract

Antimicrobial peptides (AMPs) are key to defence against infection in plants and animals. Use of AMP mutations in *Drosophila* has now revealed that AMPs can additively or synergistically contribute to defence *in vivo*. However, these studies also revealed high specificity, wherein just one AMP contributes an outsized role in combatting a specific pathogen. Here, we show the *Drosocin* locus (*CG10816*) is more complex than previously described. In addition to its namesake peptide ‘Drosocin’, it encodes a second mature peptide from a precursor via furin cleavage. This peptide corresponds to the previously uncharacterized ‘Immune-induced Molecule 7’. A polymorphism (Thr52Ala) in the Drosocin precursor protein previously masked the identification of this peptide, which we name ‘Buletin’. Using mutations differently affecting Drosocin and Buletin, we show that only Drosocin contributes to *Drosocin* gene-mediated defence against *Enterobacter cloacae*. Strikingly, we observed that Buletin, but not Drosocin, contributes to the *Drosocin* gene-mediated defence against *Providencia burhodogranariea*, including an importance of the Thr52Ala polymorphism for survival. Our study reveals that the *Drosocin* gene encodes two prominent host defence peptides with different specificity against distinct pathogens. This finding emphasizes the complexity of the *Drosophila* humoral response and demonstrates how natural polymorphisms can affect host susceptibility.

## 1. Introduction

The ability to rapidly combat pathogens is critical to organism health and survival. Organisms sense natural enemies through pattern recognition receptors, triggering the activation of core immune signalling pathways. These pathways regulate the expression of a plethora of immune effectors that provide a first line of innate defence. It was generally thought that innate immune effectors act together as a cocktail to kill microbes. However recent studies have challenged this view, revealing an unexpectedly high degree of specificity in the effector response to infection [1–3].

Chief among immune effectors are antimicrobial peptides (AMPs), host-encoded antibiotics that exhibit microbicidal activities [1,2,4,5]. Insects, and particularly the genetically tractable model *Drosophila,* have been especially fruitful in identifying and characterizing AMP potency and function [4,6–9]. In *Drosophila*, systemic infection triggers the expression of a battery of antimicrobial peptides that are secreted into the haemolymph by the fat body to transform this compartment into a potent microbicidal environment. This systemic AMP response is tightly regulated by two signalling cascades: the Toll and Imd pathways. These two pathways are similar to mammalian Toll-like receptor and tumour necrosis factor alpha/nuclear factor kappa B signalling that regulate the inflammatory response [10,11]. They are differentially activated by different classes of microbes. The Toll pathway is predominantly instigated after sensing infection by Gram-positive bacteria and fungi, while the Imd pathway is especially responsive to Gram-negative bacteria and some Gram-positive bacteria with diaminopimelic acid-type peptidoglycan [11–13]. The expression of each AMP gene is complex, receiving differential input from either pathway, with most AMPs being at least somewhat co-regulated during the systemic immune response [14–16].

In *Drosophila*, several families of AMPs contribute downstream of Toll and Imd. This includes the Cecropin, Attacin, Diptericin, Defensin, Metchnikowin, Drosomycin, Baramicin and Drosocin gene families [1,3,4]. Other host defence peptide families include Daisho and Bomanin, which are important for defence, but *in vitro* killing activity is yet to be shown [17,18]. How these immune effectors contribute individually or collectively to host defence remains poorly understood. Use of single and compound mutants has revealed that defence against some pathogens relies on the collective contributions of multiple AMP families. However, recent studies have also shown that single defence peptides can play highly specific and important roles during infection. In one case, *Diptericins* are the critical AMP family for surviving infection by *Providencia rettgeri* bacteria. This specificity is so remarkable that flies collectively lacking five other AMP gene families nevertheless resist *P. rettgeri* infection like the wild-type [6], while even a single amino acid change in one *Diptericin* gene can cause pronounced susceptibility to *P. rettgeri* [19]. Studies on Toll effector genes such as *Bomanins*, *Daishos* or *Baramicin A* have also found deletion of single gene families can cause strong susceptibilities against specific fungal species [18,20], or mediate general defences against broad pathogen types [17,21]. Lastly, loss of the gene *Drosocin* causes a specific and pronounced susceptibility to infection by *Enterobacter cloacae* [6], agreeing with Drosocin peptide activity *in vitro* [22]. Unlike the example with *Diptericins* and *P. rettgeri*, other AMPs also contribute collectively to defence against *En. cloacae* [23].

Many AMP genes encode precursor proteins with multiple peptide products processed by furin cleavage [20]. This was initially shown for the *Apidaecin* gene of honeybees, which produces nine Apidaecin peptides from a single precursor [24]. *Drosophila* also encodes many AMPs with polypeptide precursors. Examples include AMPs of the Attacin and Diptericin gene families [25,26] or *Baramicin A* which encodes three kinds of unique peptide products on a single precursor protein [1,20,27]. Meanwhile, the precursor protein of the nematode AMP ‘NLP29’ is cleaved into six similar glycine-rich peptides [28,29]. To our knowledge, the independent contributions of sub-peptides from a polypeptide AMP gene has so far never been addressed.

In this study, we reveal that the *Drosocin* gene (*CG10816*) encodes not only the antibacterial Drosocin peptide but also another host defence peptide produced by furin cleavage of the Drosocin precursor protein. We name this peptide Buletin, and show that it corresponds to IM7, an inducible peptide first identified in 1998 by MALDI-TOF analysis whose gene counterpart was never identified [30]. Using a new mutation affecting only the Drosocin peptide and not Buletin, we show that these two peptides contribute independently to defence against different microbes. Survival analyses show that while Drosocin specifically affects defence against *En. cloacae*, Buletin specifically affects defence against *Providencia burhodogranariea*. Moreover, a previously identified polymorphic site in Buletin (Thr52Ala described in [31]) mirrors the susceptibility effect of Buletin deletion to *P. burhodogranariea*. We, therefore, uncover a striking example where an AMP-encoding gene produces two peptides with distinct activities. The *Drosocin* gene is also an example of how an AMP polymorphism can significantly affect the host defence against a specific microbe. Alongside recent findings using Diptericin and *P. rettgeri*, our results highlight how AMP evolution is probably driven by differential activity against ecologically relevant microbes.

## 2. Results

For clarity of discussion: we will use the shorthand Drc (with a ‘c’, no italics) to refer to the mature Drosocin peptide. Whenever possible, we will use ‘*Drosocin* gene*’* to refer to the genomic locus (common shorthand *Dro*, with an ‘o’, italicized).

### (a) The *Drosocin* gene encodes IM7

**Figure 1.**
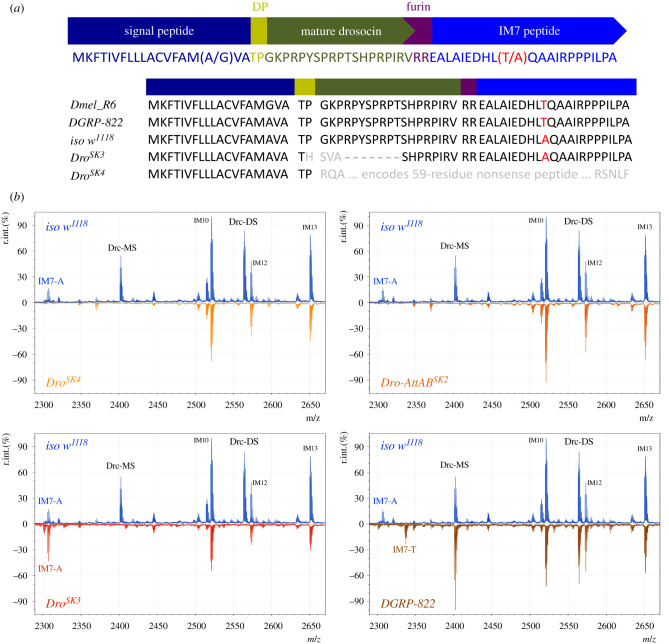
The *Dro* gene encodes a polypeptide including both Drc and IM7. (*a*) Overview of the precursor protein structure of the *Dro* gene. The Thr52Ala polymorphism in IM7 was noted previously [31]. Here we include an alignment of the Drosocin precursor protein between the Dmel_R6 reference genome and sequences from *iso w^1118^, Dro^SK3^*, *Dro^SK4^* and *DGRP-822* flies. "DP" = dipeptidyl peptidase cleavage motif. "furin" = furin cleavage site motif. (*b*) MALDI-TOF proteomic data from immune-challenged flies shows that both Drc (Drc-MS, Drc-DS) and the 2307 Da peak of IM7 is absent in *Dro^SK4^* and *Dro-AttAB^SK2^* flies. The frameshift present in *Dro^SK3^* removes the Drc peptide, but does not prevent the secretion of IM7. Threonine-encoding IM7 appears in DGRP-822 (2337 Da), alongside loss of the 2307 Da peak. (Online version in colour.)

Previous proteomic analyses of haemolymph from infected *Drosophila* revealed several immune-induced molecules (IMs) [30]. These molecules were annotated as IM1-IM24 according to their mass, and over time each of these IMs was associated with a host defence peptide gene [17,18,20,32]. At this point, only one of the 24 original IMs remains unknown: IM7. Previous efforts were unable to link this 2307 Da peptide to a gene in the *Drosophila* reference genome. However, during our studies, we noticed that IM7 was absent in flies lacking 14 AMP genes, indicating that it is probably produced by one of these genes [6,23]. We repeated these MALDI-TOF proteomic experiments with haemolymph samples from flies carrying systematic combinations of AMP mutations, ultimately honing in on the gene *Drosocin (Dro)*. Two independent *Dro* gene mutants (*Dro^SK4^* and *Dro-AttAB^SK2^*) both lack IM7 in MALDI-TOF peptidomic analysis (figure 1).

The *Dro* gene was initially identified as a single open reading frame gene encoding the Drc peptide. Drc is an O-glycosylated Proline-rich peptide that binds bacterial DnaK/Hsp70 similar to other Proline-rich insect AMPs [22,33–36]. Mature Drc requires O-glycosylation for activity, which involves the biochemical linking of either mono- (MS), di- (DS), or rarely tri-saccharide (TS) groups to the Threonine at position 11 of the Drc peptide [22,32]. These different O-glycosylations yield peptides with different mature masses of 2401, 2564, and 2767 Da (Drc-MS, -DS and -TS, respectively). Unmodified Drc peptide has an expected mass of 2199 Da, which is not an intuitive match for the 2307 Da peak of IM7, even considering other post-translational modifications. This suggests that another element of the *Dro* gene encodes IM7.

### (b) IM7 is the C-terminus product of the Drosocin precursor protein

It is puzzling that IM7 could not be annotated to the *Dro* gene given that the nucleotide sequence has been known for decades. One previous study noted that the *Dro* gene was probably cleaved at a furin-like cleavage site, and had a small undescribed C-terminal peptide [25]. Lazzaro & Clark [31] further described a polymorphism in the *Dro* gene encoding either a Threonine or Alanine at residue 52 in the C-terminus of the precursor protein sequence (Thr52Ala). The *Drosophila melanogaster* reference genome encodes the Threonine version of this polymorphism. Using the sequence of the reference genome, the Drosocin precursor C-terminus mature mass would be 2337 Da without considering post-translational modifications. If we instead substitute an Alanine at this site, the predicted mass of the Drosocin precursor C-terminus becomes 2307 Da, exactly matching the observed mass of IM7. We confirmed that our wild-type DrosDel isogenic genetic background encodes an Alanine allele both by Sanger sequencing and liquid chromatography-mass spectrometry proteomics. We next performed MALDI-TOF on the haemolymph of flies from DGRP strain 822 *(DGRP-822)*, which encodes a Threonine in its C-terminus. Exactly matching prediction, *DGRP-822* flies lack the 2307 Da IM7 peak, and instead have a 2337 Da peak that appears after infection (figure 1*b*).

Serendipitously, while generating *Dro* gene mutants using CRISPR-Cas9 we recovered a complex aberrant locus (*Dro^SK3^*) that disrupts 11 amino acid residues of the mature Drc peptide, including its critical O-glycosylated Threonine (figure 1*a*). However the *Dro^SK3^* deletion later continues in the same reading frame, including the RVRR furin cleavage site and C-terminus. Thus we suspected that the C-terminal peptide would be secreted normally in *Dro^SK3^* flies. When we ran MALDI-TOF analysis on immune-induced haemolymph from *Dro^SK3^* flies, we recovered a signal that all but confirmed the identity of the *Dro* gene C-terminus: *Dro^SK3^* flies lacked the Drc-MS and Drc-DS peaks, but the 2307 Da peak corresponding to IM7 remained immune-inducible (figure 1*b*).

Taken together, we reveal that the *Dro* gene encodes two peptides: Drc and IM7, which are produced from a precursor protein by cleavage at a canonical furin cleavage site. IM7 is a 22-residue peptide with a net anionic charge (−1.9 at pH = 7) that does not share overt similarity with Drc (+5.1 at pH = 7), though both peptides are Proline-rich. A naturally occurring polymorphism previously obscured the annotation of IM7 as a *Dro* gene product. This analysis was greatly facilitated by the use of newly available AMP mutations. We name this C-terminal peptide Buletin (Btn) after Philippe Bulet, whose dedicated efforts in the 1980s–1990s characterized many of the *Drosophila* AMPs including *Drosocin* [4,22,37].

### (c) Drosocin, but not Buletin, is responsible for the *Drosocin* gene-mediated defence against *Enterobacter cloacae*

Previous studies have suggested that flies lacking just the *Dro* gene can resist infection by most bacteria, but are specifically susceptible to infection by *En. cloacae* [6], and also somewhat *Escherichia coli* [38] and *P. burhodogranariea* [6]. The fact that the *Dro* gene encodes not one but two peptides raises the question of the specific contribution of these two peptides to previously observed *Dro* gene effects. Therefore, we took advantage of the *Dro^SK3^* and *Dro^SK4^* mutations that differently affect the Drc and Btn peptides (figure 1*a*) to explore the respective role(s) these peptides play by comparing the survival of these mutants to different infections. We focused our screen on a panel of Gram-negative bacteria of interest: *En. cloacae β12* bacteria that *Dro* gene mutants are specifically susceptible to [6,23], a recently-isolated *Acetobacter* sp*.* that can kill AMP mutant flies [39], *E. coli 1106* suggested to be affected by the *Dro* gene [22,38]*,* and *P. burhodogranariea* *strain B* where the *Dro* gene was shown to contribute to defence alongside other AMPs [6]*.* All experiments were performed with wild-type and mutant flies that were isogenized in the DrosDel genetic background according to Ferreira *et al*. [40].

**Figure 2.**
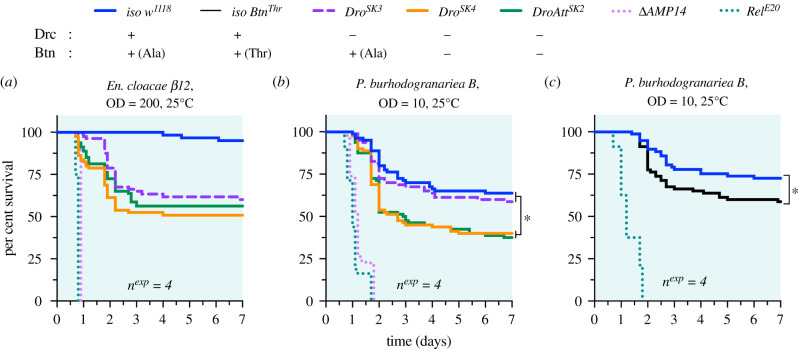
Mutations affecting Buletin cause a specific susceptibility to *P. burhodogranaria*. (*a*) *Dro^SK3^* flies succumb to infection by *En. cloacae* slightly later than either *Dro^SK4^* or *Dro-AttAB^SK2^* flies that lack both Drc and Btn. The ultimate rate of mortality is comparable (*p* > 0.05 in comparison between these various *Dro* mutants). (*b*) *Drosocin* mutants that retain Btn (*Dro^SK3^*) survive infection by *P. burhodogranariea* better than flies lacking both Drc and Btn (*Dro^SK4^, DroAtt^SK2^*). (*c*) Wild-type flies with the Threonine allele of the Btn Thr52Ala polymorphism phenocopy the effect of Btn deletion compared to Alanine-encoding *iso w^1118^* in defence against *P. burhodogranariea*. OD, optical density. (Online version in colour.)

We found that individual *Dro* gene mutants (both *Dro^SK3^* and *Dro^SK4^*) were not overtly susceptible to infection by *E. coli 1106* or *Acetobacter* sp*. ML04.1* (electronic supplementary material, figure S1). We could also repeat our previous findings that *Dro^SK4^* and *Dro-AttAB^SK2^* flies were highly susceptible to *En. cloacae* infection, causing 40–50% mortality by 3 days after infection. Importantly, use of *Dro^SK3^* flies that lack Drc but produce Btn confirms that this susceptibility is principally caused by a loss of Drc peptide and not Btn (figure 2*a*): *Dro^SK4^* and *Dro-AttAB^SK2^* flies lacking both Drc and Btn were only slightly more susceptible than *Dro^SK3^* flies lacking Drc alone, a difference that was not statistically significant (*Dro^SK4^* and *Dro-AttAB^SK2^* comparisons to *Dro^SK3^*, *p* > 0.05 in both cases).

Thus, comparison of mutants lacking Drc, or both Drc and Btn, reveals that the *Dro* gene-mediated defence against *En. cloacae* is specifically mediated by the Drc peptide. Meanwhile, Btn does not seem to contribute to defence against this bacterial infection in a significant way.

### (d) Buletin, but not Drosocin, is important for survival to *Providencia burhodogranariea* infection

We previously found that the *Dro* gene could contribute to defence against *P. burhodogranariea* synergistically alongside *Diptericins* and *Attacins* [6]. We next assessed the contribution of our different *Dro* gene mutants to defence against *P. burhodogranariea*. To our surprise, the presence or absence of Btn causes a pronounced survival difference after infection by *P. burhodogranariea*: *Dro^SK3^* flies that still produce Btn survive as wild-type, while *Dro^SK4^* or *Dro-AttAB^SK2^* flies suffer significantly increased mortality (figure 2*b*). This trend is the opposite of what is observed after infection with *En. cloacae*: Drc does not play an important role in defence against *P. burhodogranariea*, but Btn does. As emphasized by the greater susceptibility of AMP-deficient *ΔAMP14* and Imd-deficient *Rel^E20^* flies (figure 2*b*), Btn deficiency explains only part of the susceptibility to *P. burhodogranariea*. This is consistent with our previous study, which showed that the *Dro* gene contributes to defence against this bacterium alongside the contributions of *Diptericin* and *Attacin* genes.

Collectively, our study shows that the *Dro* locus encodes two host-defence peptides with distinct activities *in vivo*. This reinforces the notion that innate immune effectors can have very specific roles *in vivo*.

### (e) The Thr52Ala polymorphism affects Buletin activity against *Providencia burhodogranariea in vivo*

The existence of a Threonine/Alanine polymorphic residue in Btn in natural fly populations suggests an arms race between Btn and naturally occurring pathogens. Such polymorphisms are common in AMP genes, and are proposed to reflect host-pathogen coevolutionary selection [41,42]. The *P. burhodogranariea* strain used in this study was originally isolated from the haemolymph of wild-caught flies [43], suggesting it is an ecologically relevant microbe to *D. melanogaster*. This prompted us to investigate the contribution of this polymorphism in defence against *P. burhodogranariea*. We next isolated a Btn-Threonine allele (*Btn^Thr^*) that we introgressed into the DrosDel background over seven generations. We infected isogenic *Btn^Thr^* and *Btn^Ala^* (i.e. *iso w^1118^*) flies with *P. burhodogranariea* to determine if the Btn polymorphism impacts survival. In these experiments, *iso Btn^Thr^* flies suffered an approximately 15% increase in mortality compared to *iso w^1118^* flies with *Btn^Ala^* (figure 2*c*, *p* = 0.037). The Cox survival hazard ratio is a measure of effect size. The hazard ratio of *Dro^SK4^* versus *Dro^SK3^* flies (figure 2*b*) and *iso Btn^Thr^* versus *iso w^1118^* (figure 2*c*) is nearly-identical (hazard ratios: *Dro^SK4^-Dro^SK3^* = 0.590, *Btn^Thr^-iso w^1118^*: = 0.584). Thus the effect size of changing the Btn allele from Alanine to Threonine causes the same hazard ratio difference as the effect of Btn deletion.

We, therefore, uncover an important role of Btn in defence against *P. burhodogranariea*, and reveal that the Btn Thr52Ala polymorphism impacts survival against this ecologically relevant pathogen. Alongside the effect of a polymorphism in Diptericin on survival to *P. rettgeri* [19], here we provide a second example of how a polymorphic residue in an AMP gene significantly impacts survival.

## 3. Discussion

Here we show that the *Dro* gene encodes two peptides with distinct activities *in vivo*. Buletin was not annotated previously owing to a polymorphism that masked the identity of this second peptide. Most immune studies have used *Drosophila* strains that encode the *Btn^Ala^* allele (e.g. *Oregon-R* [30], *w^1118^* [44], *DrosDel* [6] or *Canton-S* [25]), while the *D. melanogaster* reference genome encodes the *Btn^Thr^* allele. The *Dro* gene produces a precursor protein cleaved in two locations: (i) after the signal peptide at a two-residue dipeptidyl peptidase site that is nibbled off of the N-terminus of mature Drc (electronic supplementary material, figure S3, similar sites noted in [20,45]); and (ii) at a furin cleavage motif that separates the Drc and Btn peptides (‘RVRR’ in the Drosocin precursor protein). Both cleavage motifs are common in AMPs, including *Drosophila* Attacins, Defensins, Diptericins and Baramicins, which all encode mature peptides separated by furin cleavage sites [1,20,25].

The *Dro* gene is restricted to the genus *Drosophila* [46]. However phylogenetic inference for AMPs is difficult owing to their short size [3,47], and functional analogues of the Drc peptide that may share an evolutionary history are described in many holometabolous insects [36,48]. It is therefore noteworthy that the range of Buletin is far more restricted: Buletin-like peptides are found only in *Dro* genes of fruit flies ranging from the Melanogaster to Obscura groups, and not in outgroup *Drosophila* species (electronic supplementary material, figure S2). The Buletin peptide is therefore an evolutionary novelty of the *Dro* gene C-terminus. The Thr52Ala polymorphism in Buletin is probably maintained by balancing selection [42], similar to a specific susceptibility for the Arginine variant of a Serine/Arginine polymorphism in *Diptericin* for defence against *P. rettgeri* [19]. The reason behind these polymorphisms is unclear but could rely on trade-offs in immune defence and other functions [2,49]. Trade-offs have been especially well characterized in the fly cellular immune response where higher haemocyte numbers improve host resistance to parasitoid wasps but reduce larval competitive ability [50,51]. This is probably owing to the high metabolic cost imposed by higher haemocyte numbers, reducing available fat body lipid stores [52]. Another example is the trade-off between reproduction and immunity, as both sides impose a high metabolic demand on the insect fat body in females [53–55]. While there is renewed attention on how positive selection promotes AMP polymorphisms, we know less about the evolutionary forces that maintains these alternate alleles [41,56,57]. A simple interpretation for why AMP polymorphisms exist might be that alternate residues improve resistance against specific pathogens, resulting in the maintenance of two alleles with different pathogen-specific competences. This is tempting to speculate given the *Providencia* species used here and in earlier *Drosophila* studies were isolated from wild flies [43]. Likewise, *En. cloacae* has sometimes been recovered in the microbiome of *D. melanogaster* [58]. However these proposals lack conclusive proof, as the precise logic driving the DptA or Btn polymorphisms is currently defined only by one residue being better for defence against one specific *Providencia* bacteria. Currently, we have no evidence for an alternate allele to promote defence against another pathogen. Moreover, we cannot exclude that these polymorphisms could relate to AMP roles beyond infection, as recent studies have found surprising roles for AMPs in things like memory formation and behavioural regulation [28,59–62]. For now, the evolutionary purpose of the DptA and Btn alternate alleles remains unknown.

The Drc and Btn peptides are not homologous, although both are rich in Proline residues. However, Drosocin is O-glycosylated and has a strong cationic charge (+5.1 at pH = 7), while Buletin is unmodified and has a net anionic charge (−1.9 at pH = 7). AlphaFold predicts Buletin to have an α-helical structure [63]. We screened for Buletin activity *in vitro* diluted in Luria Broth (LB) according to Wiegand *et al*. [64]. However, in our conditions, we found no effect of Buletin using either Btn^Thr^ or Btn^Ala^ against *P. burhodogranariea* or *E. coli*, even when co-incubated with sub-lethal concentrations of Cecropin (Sigma) (electronic supplementary material, figure S4). It is possible that Buletin contributes to host defence alongside a cofactor, or protects the host from a virulence factor secreted by *P. burhodogranariea*. It may even be that Buletin is required for some role in physiology unrelated to direct bacteria killing, as the *Dro* gene is expressed in a variety of epithelial tissues including the trachea [38,65]. However, we do not wish to rule out a direct action of Btn on bacteria, as our *in vitro* conditions could have been sub-optimal for revealing an antimicrobial effect. For instance, an anionic AMP of the greater wax moth synergizes with lysozyme to kill *E. coli* [66], and AMPs can act synergistically *in vitro* through complimentary mechanisms of action [26,35,67,68]. While *in vitro* approaches are a powerful demonstration for AMP function, we are realizing more and more that this is not sufficient to understand peptide activity *in vivo*. For example, the activity of azithromycin antibiotic changes 64-fold if tested in standard *in vitro* conditions or with the addition of human serum [69]. In *Tenebrio* beetles, the AMP Tenecin-2 lacks activity against *Staphylococcus aureus in vitro*, but knock down via intrathoracic injection of double stranded RNA causes a significant mortality to *S. aureus* infection *in vivo* [70]. In *Drosophila*, Bomanin peptides do not display activity *in vitro*, but Bomanin-deficient haemolymph loses Candida-killing activity [21]. While AMPs were first identified for their potent microbicidal activity *in vitro* [4,9,71], recent studies in *Drosophila* have recovered striking specificity of AMPs in defence *in vivo* that was never predicted from *in vitro* analyses [6,18,19]. These results suggest both *in vitro* and *in vivo* approaches are necessary to shed light on host defence peptide activity.

It is striking that the Threonine/Alanine polymorphism in Buletin affects the fly defence against *P. burhodogranariea*. This polymorphism is found in wild populations of *D. melanogaster*, and at high frequencies in the Drosophila Genetic Reference Panel: 29% Threonine, 64% Alanine, 7% unknown at DGRP allele 2R_10633648_SNP [31,72]. A polymorphism in *Diptericin A* causes a profound susceptibility to defence against *P. rettgeri* [19], and similar polymorphisms are found in various AMP genes of flies [41,42] and other AMP genes from animals including fishes, birds, and humans [56,73,74]. We now add our study on Buletin and *P. burhodogranariea* to the building evidence that such polymorphisms can have major impacts on microbial control. In other species, AMP polymorphisms could have important implications on immune competence of individuals or groups. For instance: we might wonder if inbreeding in honeybees could have fixed disadvantageous AMP alleles contributing to colony collapse disorder [75]. Reduced AMP expression is also associated with conditions like psoriasis [76] or susceptibility to *Pseudomonas aeruginosa* infections [77,78]. A targeted screen has even suggested polymorphisms in human *ß-Defensins* correlate with atopic dermatitis [79]. Could polymorphisms in human AMPs help explain predisposition to similar infectious syndromes?

## 4. Conclusion

By uncovering a novel host defence peptide, our study contributes to a growing body of literature establishing the *Drosophila* systemic infection model as boasting the unique ability to reveal specific interplay of host effector-pathogen interactions. This mode of infection allows the use of the fly haemolymph as an arena to monitor pathogen growth in the presence of effectors, with fly survival as a rapid readout. While previous studies *in vitro* have suggested fly AMPs had generalist activities, use of specific mutations affecting individual AMP genes has now revealed specific relationships between host and pathogen. Early *in vitro* studies would never have predicted the highly specific requirement for only single peptides in defence against specific pathogens. Taking lessons from the fly, it should be of significant interest to characterize the differential activity of AMP polymorphisms in humans and other animals, which could reveal critical risk factors for infectious diseases.

## 5. Material and methods

### (a) Fly genetics

Genetic variants were isogenized into the DrosDel isogenic background over seven generations as described in [40]. The specific mutations studied here were sourced as follows: the *Dro^SK3^* mutation was generated by CRISPR-Cas9 via gRNA injection as described in [80]. The *Dro^SK3^* sequence was validated by Sanger sequencing and the nucleotide and translated sequence is shown in the electronic supplementary material, figure S3A. *Dro^SK3^* flies encode a truncated version of the Drc peptide lacking its critical Threonine needed for O-glycosylation, and we could detect variants of this truncated Drc peptide in MALDI-TOF spectra with variable degradation of the N-terminus (electronic supplementary material, figure S3A-B). The *Btn^Thr^* allele used in this study was originally detected in *Def^SK3^* flies from Parvy *et al*. [81] by virtue of mutation-specific MALDI-TOF proteomics while screening for possible source genes of IM7. After isogenization, *iso Btn^Thr^* flies were confirmed to have a wild-type *Defensin* gene by polymerase chain reaction. Sequence comparisons were made using Geneious R10.

### (b) Microbe culturing conditions for infections

Bacteria were grown to mid-log phase shaking at 200 r.p.m. in their respective growth media (Luria Bertani, MRS + Mannitol) and temperature conditions, and then pelleted by centrifugation to concentrate microbes. Resulting cultures were diluted to the desired optical density (OD) at 600 nm for survival experiments, which is indicated in each figure. The following microbes were grown at 37 °C: *E. coli strain 1106* (LB)*, P. rettgeri* (LB)*.* The following microbes were grown at 29°C: *P. burhodogranariea* (LB) and *Acetobacter* sp*. ML04.1* (MRS + Mannitol).

### (c) *In vitro* antibacterial assays

Both the Btn^Thr^ and Btn^Ala^ versions of the 22-residue IM7 peptide were synthesized by GenicBio to a purity of greater than 95%, and silk moth Cecropin A was provided by Sigma-Aldrich at a purity of greater than or equal to 97%. Peptide preparations were verified by high performance liquid chromatography. Peptides were dissolved in water, and concentrations verified by a combination of bicinchoninic acid assay and Nanodrop A205 readings alongside a bovine serum albumin standard curve. We screened Btn for activity against both *P. burhodogranariea* and *E. coli* alone at 100 µM–1 mM, or at 100 µM in combination with serially diluted Cecropin concentrations spanning the Cecropin MIC (10 µM–0.1 µM). Microbes were allowed to grow to log-growth phase, at which point they were diluted to OD = 0.0005 in LB, and then 80 µl of this dilute culture was added to 20 µl of water or peptide mix to reach desired concentrations in a 96-well plate. Bacteria-peptide solutions were left overnight at room temperature and checked for growth the next morning, and in one experiment OD at 600 nm was recorded every 10 min using a TECAN plate reader (electronic supplementary material, figure S4).

Using these conditions, we found a minimum inhibitory concentration (MIC) for Cecropin A against *E. coli 1106* of approximately 1 µM, agreeing with previous *E. coli* literature [82]. We found an MIC of Cecropin A against *P. burhodogranariea* of approximately 5 µM, though even 0.63 µM delays growth by approximately 3 h compared to no-peptide controls (electronic supplementary material, figure S4). Even at 1 mM, neither the Btn^Thr^ nor Btn^Ala^ showed any growth inhibition alone, and 100 µM peptide combinations with Cecropin A showed no reduction of MIC over Cecropin A alone. 100 µM represents the upper limit of AMP concentration in fly haemolymph after infection [83], and the concentration of Btn *in vivo* is probably much lower than this based on MALDI-TOF relative peak intensities [6,20,30,32]. As we tested Btn alone at 1 mM, and at 100 µM Btn + Cecropin across the Cecropin MIC range, we find that at least in our conditions using LB as diluent, Btn does not display *in vitro* activity.

### (d) Survival experiments

Survival experiments were performed as previously described [6], with 20 flies per vial with total replicate experiment number reported within figures (*n^exp^*). Approximately 5-day old males were used in experiments, pricked in the thorax at the pleural sulcus. Flies were flipped thrice weekly. Statistical analyses were performed using a Cox proportional hazards (CoxPH) model in R 3.6.3.

### (e) Proteomic analyses

Raw haemolymph samples were collected from immune-challenged flies for MALDI-TOF proteomic analysis as described previously [6,30]. In brief, haemolymph was collected by capillary and transferred to 0.1% trifluoroacetic acid before addition to the acetonitrile universal matrix. Representative spectra are shown. Peaks were identified via corresponding *m*/*z* values from previous studies [20,32]. Spectra were visualized using mMass, and figures were additionally prepared using Inkscape v. 0.92.

## Data Availability

All data are available within the manuscript and the electronic supplementary material [84].
